# Alveolar deflation dynamics before and after lung injury assessed by synchrotron radiation computed tomography

**DOI:** 10.1186/2197-425X-3-S1-A567

**Published:** 2015-10-01

**Authors:** G Perchiazzi, JB Borges, G Hedenstierna, L Porra, L Broche, M Pellegrini, A Sindaco, AP Tannoia, S Derosa, FFM Todisco, G Scaramuzzo, A Larsson, S Bayat

**Affiliations:** Bari University, Emergency and Organ Transplant, Bari, Italy; Uppsala University, Surgical Sciences, Uppsala, Sweden; Uppsala University, Medical Sciences, Uppsala, Sweden; University of Helsinki, Physics, Helsinki, Finland, Finland; European Synchrotron Radiation Facility, Grenoble, France; Université de Picardie Jules Verne, Laboratoire Peritox EA-INERIS, Amiens, France

## Introduction

During mechanical ventilation, reduction of expiratory pressure may trigger heterogeneous processes of derecruitment, collapse and overdistension of airspaces. However the relationship of such processes with precise regional location inside the lung has not been fully studied *in vivo*. Synchrotron Radiation Computed Tomography (SRCT) can provide *in vivo* regional images of the lung at resolutions higher than conventional CT.

## Objective

To evaluate regional deflation dynamics in healthy (HC) and Acute Respiratory Distress Syndrome (ARDS) conditions.

## Methods

Seven New Zealand rabbits were anesthetized and mechanically ventilated with a tidal volume of 7 ml/kg and positive end-expiratory pressure (PEEP) 3 cmH_2_O. We studied airspaces located in three concentric regions-of-interest (ROI): subpleural (SP), peripheral (PE) and core (CO). During expiratory pauses, SRCT scans of the lung were taken at decreasing PEEP levels of 12, 9, 6, 3 and 0 cmH_2_O. Then, ARDS model was established by repeated lung lavages followed by ventilator-induced lung injury (by pressure-controlled ventilation with inspiratory pressure of 35 and PEEP = 0 cmH_2_O). SRCT scans at the same decremental PEEP levels were repeated. SRCT images with a spatial resolution of 47.6 µm were enhanced by phase contrast algorithms, transformed into binary images, and divided into the above-defined ROIs. Numerosity (N), total area (A), and Area/Numerosity ratio (A/N) indexes were computed by using the Image Processing Toolbox for MatLab (Mathworks, Natick, USA). Statistical analyses to test differences produced by PEEP and/or location were performed by Student's T-test (α = 0.05).

## Results

In HC, N and A decreased with decreasing PEEP, which was more evident in CO. A/N remained stable down to PEEP 9 cmH_2_O and then decreased with PEEP. In ARDS, N and A decreased down to PEEP 6 cmH_2_O and then remained stable at subsequent lower PEEP levels. SP, PE and CO areas exhibited similar behaviors but with different magnitudes. In ARDS, between PEEP 12 and PEEP 6 cmH_2_O, A/N index behavior evidenced that airspaces dimensions in CO were stable, but decreased below PEEP 6 cmH_2_O. At each PEEP level, mean dimensions of airspaces were greater in CO than in SP regions.

## Conclusions

In healthy conditions, derecruitment occurred continuously along the decremental PEEP levels. When airspaces were fully inflated, they exhibited a more stable behavior, losing less volume. Reducing PEEP from 9 to 0 cmH_2_O, derecruitment was proportionally greater in the more inflated airspaces, which were predominantly located in the core regions. In ARDS, loss of gas volume between PEEP 12 and 6 cmH_2_O was characterized by an “on-off mechanism” of derecruitment, remaining stable the dimensions of the still open airspaces. This may be related to the higher critical closing pressures and lower compliance of ARDS lungs.

## Grants

The School of Anesthesia, Bari University; The Swedish Research Council; The Heart Lung Fund; The ESRF.

